# A Small and High-Speed Driving Mechanism for 3D Shape Measurement in Monocular Endoscopy

**DOI:** 10.3390/s21144887

**Published:** 2021-07-18

**Authors:** Yoshikazu Nakajima, Nobuyuki Tanigaki, Takaaki Sugino, Toshihiro Kawase, Shinya Onogi

**Affiliations:** 1 Department of Biomedical Information, Institute of Biomaterials and Bioengineering, Tokyo Medical and Dental University, Tokyo 101-0062, Japan; sugino.bmi@tmd.ac.jp (T.S.); kawase.bmi@tmd.ac.jp (T.K.); onogi.bmi@tmd.ac.jp (S.O.); 2 Department of Bioengineering, Graduate School of Engineering, The University of Tokyo, Tokyo 113-8656, Japan; tanigaki@nakajimalab.org

**Keywords:** three-dimensional shape measurement of endoscopy, shape from focus, small driving mechanism for shape from focus

## Abstract

Three-dimensional (3D) shape acquisition has been widely introduced to enrich quantitative analysis with the combination of object shape and texture, for example, surface roughness evaluation in industry and gastrointestinal endoscopy in medicine. Shape from focus is a promising technique to measure substance surfaces in 3D space because no occlusion problem appears in principle, as does with stereo shape measurement, which is another commonly used option. We have been developing endoscopic shape measurement devices and shape reconstruction algorithms. In this paper, we propose a mechanism for driving an image sensor reciprocated for the shape from focus of 3D shape measurement in monocular endoscopy. It uses a stepping motor and a planar-end cam, which transforms the motor rotation to imaging sensor reciprocation, to implement the shape from focus of 3D shape measurement in endoscopy. We test and discuss the device in terms of its driving accuracy and application feasibility for endoscopic 3D shape measurement.

## 1. Introduction

Three-dimensional (3D) shape acquisition has been widely developed for industries and research fields in the last decade. In industrial production, 3D shape reconstruction can be introduced to evaluate the roughness of surface decoration and sliding joints. In the medical field, it is efficient for diseased area detection in gastrointestinal endoscopy due to the consideration of both surface shapes and textures and works better than conventional 2D image endoscopy. Optical coherence tomography shows good performance in acquiring image volume around tissue surfaces [1,2,3]. It may be being introduced into practical clinics, but it still has a drawback on scanning time for sweeping the spot in the measurement volume. Although some researchers have addressed the acquisition of speed acceleration, it might not be enough to measure the whole volume in appropriate time periods. In addition, a small area of measurement might be another drawback for endoscopic applications. Confocal laser endomicroscopy is another concern for this purpose [4,5], but the device might still be larger than the specification to be introduced into gastrointestinal tracts. For technological concerns, many approaches to capture 3D object shapes have been proposed. Stereo measurement with two or multiple cameras is employed in many cases [6,7]. It is one of the main ways but internalizes ambiguity on the correspondence of two-sight object textures. The combination of laser-beam pattern projection and camera capturing can fix the problem but might be unstable because of a change in the reflection power of the laser beam with respect to the material condition at each point of the tissue surface. Moreover, stereo measurement methods cause occlusion at some of the measurement volume. Shape from shading [8,9] is another option to obtain a shape but requires Lambertian reflectance of object surfaces. The constraint of Lambertian reflectance might, however, be hard to apply to medical applications due to the surface texture and heterogeneous ray reflectance of most organs. Shape from motion [10,11] is an option but would require convergence angles among multiple cameras and a high number of captured images. The need for camera motion would hamper the smooth operation of endoscopic surgical treatment. Shape from focus and defocus [12,13,14] (SFF and SFD) might be a promising way to capture the 3D shape of organs because of the utilization of textures appearing on them. As SFF measures with a single optical system, it does not need to find pixel correspondence. Thus, no occlusion appears in the measurement volume. In addition, single optical systems are good for reducing the device size compared with stereo measurement devices. The advantage relative to shape from shading is high accuracy and stability because of the rich texture of the soft organ surface. The advantage relative to shape from motion is that there is no need to move the camera position. Therefore, we have proposed a method to reconstruct object shape from focus-controlled image sequences and have developed a prototype device for it [15]. It worked well for laparoscopy due to the camera location outside the patient’s body, but there might have been a problem with the device size for endoscopy application. To improve SFF accuracy and stability, many software algorithms have addressed noise reduction [16,17,18] and point-spread-function optical modeling [19]. In addition, SFF hardware imagers have been proposed [20,21], but have not been introduced for endoscopy. Takeshita et al. proposed endoscopic SFF, but the hardware was still 150 mm in length with a 15 mm diameter [15]. The rigid part of the 150 mm length can hamper bending the endoscope shaft for smooth insertion into the body. In this paper, we propose a small and high-speed driving mechanism for shape measurement in endoscopy with SFF. The device prototype is 11 mm in diameter and 23 mm in length and measures 3D shapes and textures of organs with 1 to 5 Hz measurement speed. It uses a stepping motor for driving and a magnetic-end cam for transforming the motor rotation into the imaging sensor reciprocation for SFF. Cylindrical ribbed or grooved cams are commonly used to transform rotation into reciprocation, but it is difficult to assemble the small devices due to a backlash at the concavo-convex part contact. One-end cams reduce such a backlash for pushing displacement, but a compressing mechanism needs to be introduced for the opposite directed motion that the cam pushes. A magnetic-end cam is a possible option to fulfill reciprocation driving for our purpose. Although the size of a 2D endoscopic device is 5–8 mm, our device might be one of the smallest devices able to quantitatively measure 3D organ shapes.

## 2. Method

### 2.1. Principle of Shape from Focus

**Figure 1:**
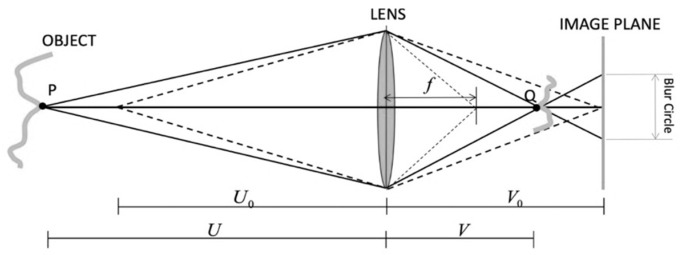
Focusing geometry of the imaging system.

The principle of SFF is noted here briefly. Let f, U, and V be the focal length of the imaging system, the distance between the lens and the focal point in the space, and the distance between the lens and the imaging point, respectively, as shown in Figure 1. U locates for the object side from the lens and V locates for the imaging-plane side. Their relationship can be expressed with the equation of

(1) $$\frac{1}{U}+\frac{1}{V}=\frac{1}{f}$$

Let $V_{0}$ be the distance from the lens to the imaging plate; Equation (2) was developed for the function that $V_{0}$ delivers the geometry of focused point U in the object space with the imaging parameter f as

(2) $$U\left(V_{0};f\right)=\frac{V_{0}f}{V_{0}-f}$$

Laplacian of Gaussian filtering might be commonly introduced to determine the focal level of each pixel as

(3) $$f_{\mathrm{focal}\;\mathrm{level}}\left(x,y\right)=LOG\left(x,y\right)*\left|h\left(x,y\right)*i\left(x,y\right)\right|$$

where x and y are the coordinates of each pixel on the image. The operation * means convolution. i(x,y) is the intensity at (x,y). h(x,y) is a point spread function, which can be denoted as a Gaussian function in most cases:

(4) $$h\left(x,y\right)=\frac{1}{2\pi \sigma^{2}}\exp\left(-\frac{x^{2}+y^{2}}{2\sigma^{2}}\right)$$

LOG(x,y) is expressed as

(5) $$LOG\left(x,y\right)=\frac{1}{\pi \sigma^{4}}\left(1-\frac{x^{2}+y^{2}}{2\sigma^{2}}\right)\exp\left(-\frac{x^{2}+y^{2}}{2\sigma^{2}}\right)$$

which works as a frequency bandpass filter, in addition to focal level determination with a second-order derivative. Maximum-point determination of $f_{\mathrm{focal}\;\mathrm{level}}\left(x,y\right)$ delivers the imaging-plane coordinate focusing, $V_{0}\left(x,y\right)$, for each pixel point (x,y). Then, $V_{0}\left(x,y\right)$ delivers the coordinate U of the object surface at each point with Equation (2). Computing optimal U for each pixel at (x,y), which means the amount of depth points, the object surface is given. In addition, sharpened texture colors and intensities are provided for each point by extracting and interpolating the images around the focused position of the image plane. Finally, the process provides a three-dimensional textured surface of the object.

### 2.2. Device Design and Prototype Assembly

**Figure 2:**
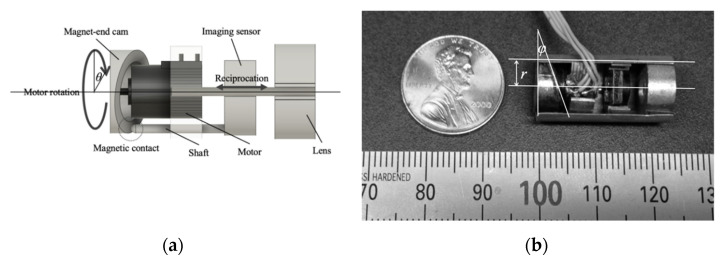
Prototype: (**a**) schematic diagram and (**b**) appearance of internal mechanism.

Three-dimensional shape reconstruction needs a mechanism that drives the image sensor and reciprocates linearly to obtain a focused and defocused image sequence. Figure 2a is a schematic diagram showing the image-sensor drive we have developed. It employs a magnet-end cam, which is driven by a stepping motor (SMH6-20, Minebea-Mitsumi Co. Ltd., Tokyo, Japan). The image-sensor drive was produced as described below. Its end was cut at a tilted plane and oblique with an angle φ. The end is made of a thin plate of neodymium magnet (NR0004, Magfine Co. Ltd., Miyagi, Japan), with dimensions of 10, 6, and 1 mm for the outside diameter, inside diameter, and thickness, respectively. In addition, it was coated with vinyl chloride to smoothen the cam motion for the shaft introduced below. An image sensor (PPV801C, Asahi Electric Laboratories Co. Ltd., Tokyo, Japan) was positioned by two linear rail sliders located on both sides. It has a one-eighth of an inch CMOS for color imaging and is 2.8 μm squared. The pixels are 640 and 480 for the horizontal and vertical directions, respectively. It was connected to an I2C bus control of a desktop computer with a 200 mm extension cable, which provided YUV 422 or RGB 565 of an 8-bit parallel signal. A shaft was fixed to the image sensor at one edge of it and contacted with the magnetic cam at the other edge. Motor rotation was propagated to the reciprocation of the image sensor, which is linear and parallel to the sight line of the imaging coordinate system, through the magnet-end cam. The prototype we fabricated is shown in Figure 2b. It has a short cylinder shape. The total dimensions are 11 mm in diameter and 23 mm in length. The weight is 4.75 g. The depth of the imaging field is 17 to 100 mm, which might be around the range of shape measurement displaced from the lens. The imaging is completed in around 0.2 s intervals. The stepping motor drives the cam and propagates its rotation to the imaging sensor as reciprocation. Let r, θ, and φ be the circular-trajectory radius of shaft-to-cam contact, as shown in Figure 2, the angle of motor driving, and the angle of cam plane tilting, respectively. The displacement, Δz, of the imaging sensor is described as

(6) Δz=rtanφ(1+cosθ)

It can be expressed with the maximum of the cam stroke, $d_{\max}$,

(7) $$\Delta z=\frac{d_{\max}}{2}\left(1+\cos\theta \right)$$

where tanφ is given geometrically as $\frac{d_{\max}}{2r}$. $d_{\max}$ is 3 mm for our device.

## 3. Experiments

### 3.1. Accuracy Test for Image-Sensor Reciprocation

**Figure 3:**
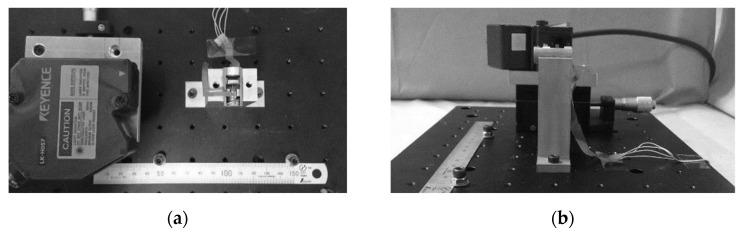
An experimental setup to measure the device’s motion accuracy: (**a**) top view and (**b**) side view.

**Figure 4:**
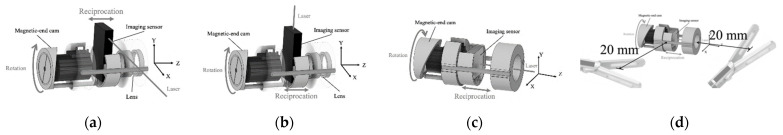
Accuracy of device motion was measured independently for the (**a**) x-axis, (**b**) y-axis, and (**c**) z-axis. The metal effect was measured with the dimensions shown in (**d**).

We tested the accuracy of reciprocation motion by the device mechanism with experimental setups, as shown in Figure 3. The three-dimensional position for the imaging sensor was evaluated using a laser displacement meter (LK-H057, Keyence Co., Ltd., Tokyo, Japan), which emits a 650 nm wavelength laser and measures an object position with 0.025 μm accuracy in 50 ± 10 mm of measurement volume. The laser displacement meter was fixed onto the X–Y stage with an aluminum jig. Three perpendicular components of x-, y-, and z-axis displacement were measured independently in imaging sensor positioning, as shown in Figure 4. The z-axis was calibrated as parallel to the sight of the imaging view. The sampling rate by the laser displacement meter was 10,000 Hz, and 600,000 points were measured in 60 s for each test trial. The device was placed for each pose of horizontal, vertically upward, and vertically downward. Measurements were taken 50 times for each axis component, each device pose, and 1 to 5 Hz of reciprocation frequency. The results were evaluated with the root-mean-squared error (RMSE) for each experimental condition. In addition, the effect of metal, such as surgical tools or devices, was evaluated. Two stainless steel surgical tools, i.e., laparoscopic forceps (ENDO GRASP^TM^, Coviden-Medtroinc Corporation, Minneapolis, MN, USA), were placed as follows: one 20 mm forward from the lens and one 20 mm sideward from the device body surface. The dimensions are shown in Figure 4d.

### 3.2. Feasibility Test for 3D Shape Measurement of Organs

**Figure 5:**
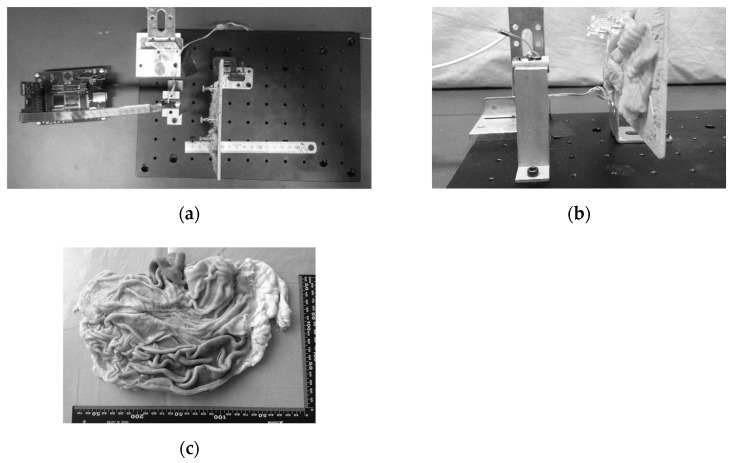
Shape measurement test with an in vitro porcine stomach. Experimental setup: (**a**) top view; (**b**) side view; and (**c**) substance.

We tested the feasibility of 3D shape measurement for organ surfaces. An in vitro porcine stomach was used as the substance for this test, as shown in Figure 5. The substance was stuck onto a wooden board with pins and placed in front of the device. The 3D shape of the substance surface was given by the device and the application feasibility was discussed.

## 4. Results

### 4.1. Accuracy of Image-Sensor Positioning in SFF Reciprocation

**Figure 6:**
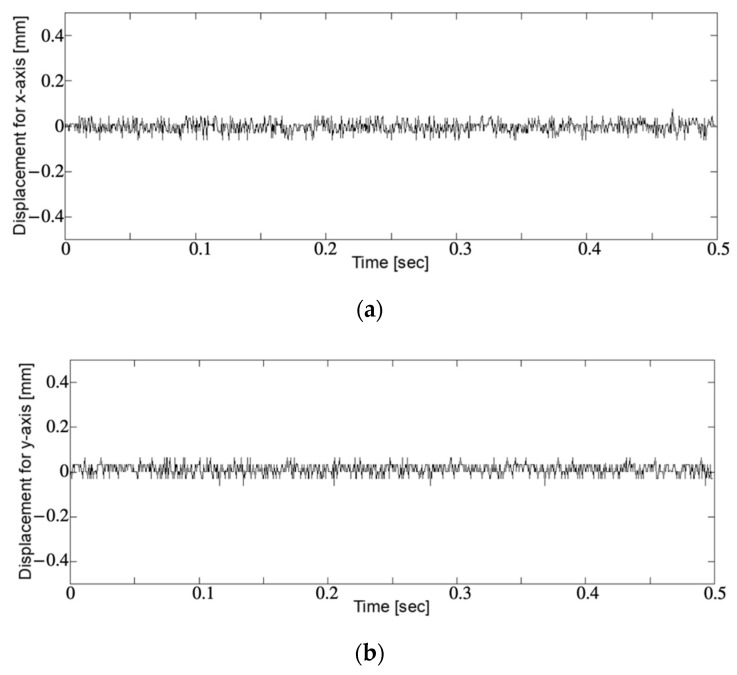

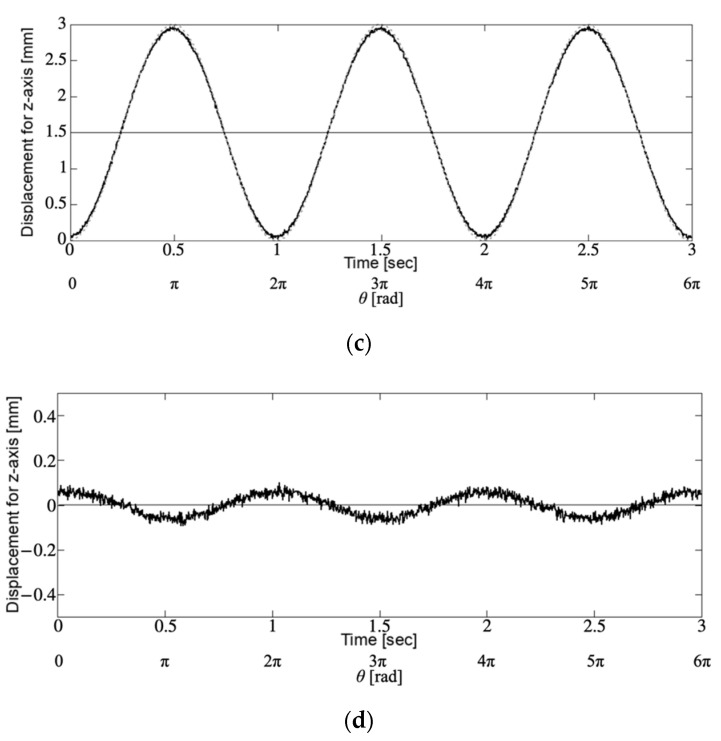
Imaging sensor displacement for (**a**) x-axis, (**b**) y-axis, and (**c**) z-axis directions. For (**a**) and (**b**), the time duration was 0.5 s. For (**c**), it was 3 s. In (**c**), the dashed curve shows the ideal points, and the curve shows the measurements by the laser displacement meter. The error of data shown in (**c**) relative to the sine wave is highlighted by (**d**). The vertical axes are in the 1 mm range for (**a**), (**b**), and (**d**), and 3 mm range for (**c**).

**Figure 7:**
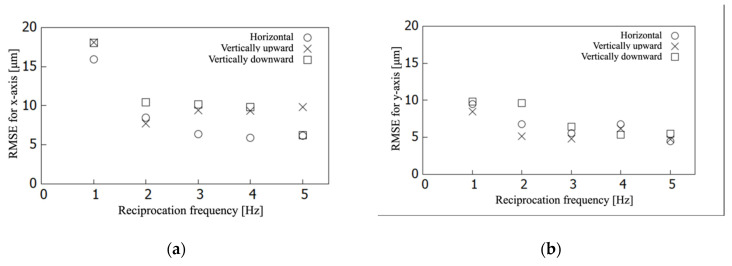

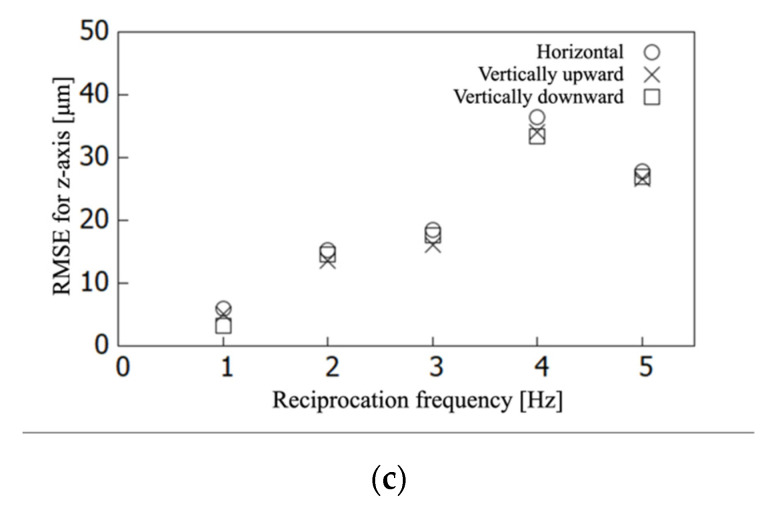
RMSEs of imaging sensor positioning for each horizontal, vertically upward, and vertically downward direction of the device pose. They are given with respect to the device reciprocation frequency for each coordinate-system component of the (**a**) x-axis, (**b**) y-axis, and (**c**) z-axis.

**Figure 8:**
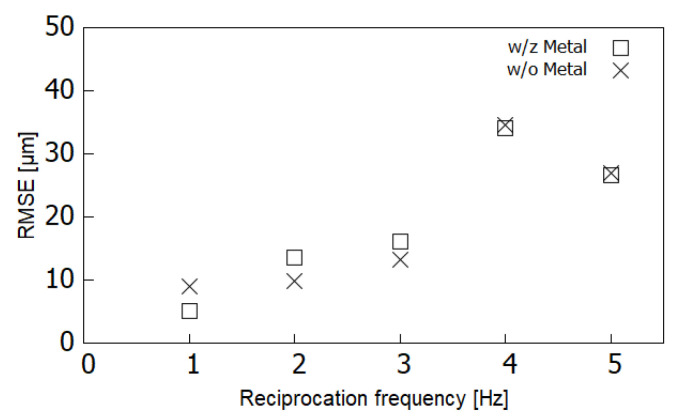
RMSEs of imaging sensor positioning with respect to the device reciprocation frequency for each condition with and without the metal placement close to the device.

Figure 6 shows the position of the imaging sensor with respect to time, cropped for 0.5 s for both the x- and y-axis and 3 s for the z-axis. The dashed curve shows the ideal points, and the curve shows the points collected by the laser displacement meter. Each component of the x-, y-, and z-axis was measured and drawn separately. The x- and y-axis components were small compared with the measurement resolution of the laser displacement meter. They were around zero level and associated with the positional perturbation of 24.3 μm RMSE. The z-axis component oscillated between 0 and 3 with 1 Hz reciprocation in the ideal situation. It showed that reciprocation driving did not attain the edge levels of 0 and 3 regarding wave amplitude but the residue RMS was 47.1 μm. Most of the errors were caused by an insufficient amplitude of reciprocation. Figure 7 shows the RMSE of imaging sensor positioning for the x-, y-, and z-axis directions with respect to the frequency of imaging sensor reciprocation for SFF. The device took each pose of horizontal, vertically upward, and vertically downward relative to ground horizontal level, which was gravitational-force direction with another expression, in the test. The RMSEs were around 10 μm for the axes perpendicular to the reciprocation sliding. For the z-axis, the axis of reciprocation sliding, the RMSE showed an approximately linear increase in the reciprocation frequency up to 40 μm at 4–5 Hz of reciprocation. Figure 8 shows the RMSE of imaging sensor positioning with respect to device reciprocation frequency for each condition with and without metal placement close to the device. It shows that device positioning was affected by the metal around the device, highlighted by the increasing reciprocation frequency. The result trended similar to the result shown in Figure 7c. For the x- and y-axis, the error was less than 20 μm and showed no obvious trend for the direction of magnetic force.

### 4.2. 3D Shape Measurement for the In Vitro Porcine Stomach

**Figure 9:**
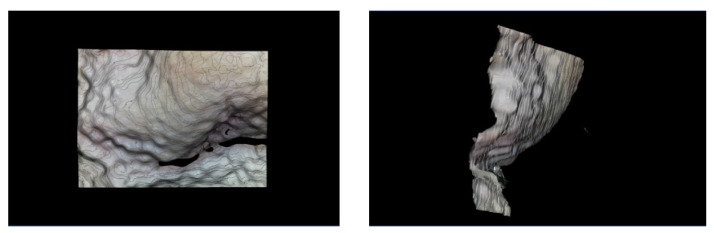
Result of textured surface acquisition for an in vitro porcine stomach.

We checked the capability of the device to provide the 3D shape of organ surfaces with the in vitro porcine stomach. The result is shown in Figure 9. It looks as though the shape and texture of the surface were given intuitively to allow for the inspection of disease on the organ surfaces. In this experiment, the sensor was positioned from 2.5 to 5.5 mm relative to the lens. Based on the results of Takeshita’s experiment using the same lens system [15] and the sensor position in our experiment, the measurement was from 11.2 to 32.1 mm and thus the measurement volume was 20.9 mm in real space. Since the shape was measured with the sensor position from 3.8 to 4.2 mm for this substance, the error of the sensor position was enlarged 4.8 times and the estimated measurement error was 0.16 mm in real space. No occlusion or irregular correspondence were observed in the result.

## 5. Discussion

Figure 6a,b shows positioning perturbation perpendicular to the reciprocation driving. It looks noisy but is not so large. It might be frictional vibration. It could be that the two linear rail sliders worked well to reduce the altitude perturbation of the imaging sensor. The result shown in Figure 6c shows an error component directed to reciprocation driving. It caused an error on the amplitude and phase of wavy curve of reciprocation. This might have been caused by some of the backlash or manufacturing error of the cam. The amplitude error appeared as gaps at the top and bottom of the wavy trajectory of the imaging sensor. No obvious perturbation appeared. Tilting of the sensor plane did not appear for this error because the z-axis component was measured at the center of the imaging sensor. Although some backlash error appeared in the measurement, the error might have been accepted to reconstruct the substance shapes because the range we used for practical measurement was at around 3.0 mm of displacement.

Figure 7a,b shows that errors for the x- and y-axis were not significant, whereas the z-axis error shown in Figure 7c increased linearly with respect to the reciprocation frequency. Some backlash would have been caused by frictional force at the rail sliders and the gravitational force added to the imaging sensor. In addition, the vinyl chloride coating to the cam surface might have slightly expanded the backlash. In addition, the results showed that the reciprocation frequency affected the driving accuracy. This might have appeared as a backlash of motion perturbation. The device was tested on accuracy with three device positionings of horizontal, vertically upward, and vertically downward. The results did not show obvious differences among them.

The result shown in Figure 8 trended similar with the result shown in Figure 7c. External force added to the device was magnetic force for Figure 8 and ground gravity for Figure 7c. The causes were different but the same as that of an external force affecting the device. The results may have shown that an external force would affect device positioning and linearly increase the frequency of device reciprocation.

The images in Figure 9 show the visualization of the condition of the porcine stomach wall, and they might be able to support disease inspection thanks to the intuitive visualization of the textured surface. Considering our clinical target of detecting 3–5 mm tumors in gastrointestinal endoscopy and laparoscopic robot surgeries, the required accuracy for shape measurement might be around 1 mm. Thus, the proposed mechanism might have worked acceptably for this application because of the RMSE being less than 0.2 mm. No occlusion or irregular correspondence were observed in the result. The proposed device is an engineering step toward endoscopic 3D shape measurement. As the device size might still be around the upper range, more precise assembling needs to be discussed. Note that clinical feasibility, including usability, has not been evaluated here and should be addressed in future research. 

## 6. Conclusions

We have proposed a mechanism that drives an image sensor reciprocated for SFF object shape measurement. The device size is 11 mm in diameter and 23 mm in length. It might be acceptable for endoscopic imaging. The results of the driving test showed that the error of the image-sensor positioning was less than 40 μm. The prototype device succeeded in providing a 3D shape of an in vitro porcine stomach. Although we should test the accuracy of the shape measurement in future research, the device showed a feasibility to capture organ shapes in endoscopy.

## Data Availability

The data presented in this study are available on reasonable request from the corresponding author.
